# Efficacy of Intravenous Thrombolysis for the Prognosis of Branch Atheromatous Disease

**DOI:** 10.7759/cureus.81301

**Published:** 2025-03-27

**Authors:** Taiki Matsubayashi, Shuko Fujiki, Ryoko Muramatsu, Misako Furuki, Masato Obayashi

**Affiliations:** 1 Department of Neurology, National Hospital Organization Disaster Medical Center, Tokyo, JPN

**Keywords:** alteplase, branch atheromatous disease, intravenous thrombolysis, modified rankin scale, national institutes of health stroke scale

## Abstract

Branch atheromatous disease (BAD) is often resistant to treatment, and the efficacy of intravenous thrombolysis (IVT) using recombinant tissue-type plasminogen activator remains uncertain. This study aimed to evaluate the effect of IVT on the prognosis of patients with BAD.

We conducted a retrospective cohort study of BAD patients who arrived at our hospital within 4.5 hours of symptom onset. Patients were divided into two groups based on treatment: the IVT group (n = 11) and the non-IVT group (n = 87). In the IVT group, alteplase (0.6 mg/kg) was administered intravenously. Clinical outcomes were compared between these groups. Additionally, within the IVT group, we performed a subgroup analysis, defining patients with a modified Rankin Scale (mRS) score of ≤2 at discharge as having a favorable outcome and those with an mRS score of ≥3 as having an unfavorable outcome.

Patients in the IVT group were significantly younger than those in the non-IVT group (62.4 years vs. 75.4 years; p = 0.0003). No significant differences were observed between the two groups in the National Institutes of Health Stroke Scale (NIHSS) scores at admission and discharge or in mRS scores at discharge. In the IVT group, patients with a favorable prognosis (n = 5) were significantly younger than those with a poor prognosis (n = 6) (53.4 years vs. 69.8 years; p = 0.0088). However, NIHSS scores at admission did not significantly differ between the favorable and poor prognosis groups. No intracranial hemorrhagic complications were observed in the IVT group.

This study found no clear benefit of IVT on the prognosis of BAD patients, underscoring the need for novel treatment strategies. Age appears to influence the prognosis of BAD patients treated with IVT, consistent with findings in ischemic stroke in general. This study had a small sample size for the IVT group and was a retrospective, single-center observational study. Therefore, a large-scale prospective randomized controlled trial is needed to evaluate the efficacy of IVT for BAD in the future.

## Introduction

Ischemic stroke presents with a variety of neurological symptoms, including motor paralysis, sensory disturbances, and ataxia, many of which often persist as long-term sequelae. As a result, ischemic stroke is a leading cause of disability and a major contributor to the need for long-term care, highlighting the importance of developing effective treatment strategies [1]. Endovascular therapy has been gaining increasing attention for the treatment of acute ischemic stroke [2].

Branch atheromatous disease (BAD)-type stroke occurs due to the atherothrombotic occlusion of the origins of deep penetrating arteries, such as the lateral striate arteries or paramedian pontine arteries [3]. The global epidemiology of BAD remains unclear; however, most reports originate from Asia [4]. In Japanese populations, BAD accounts for 9.1% of ischemic strokes [5], while in Hong Kong populations, it accounts for 20.4% [6]. The high frequency of BAD in the Asian population may be attributed to racial variations in stroke etiology. While embolic strokes, including those associated with atrial fibrillation, are more common in Caucasian populations, Asian populations exhibit a higher prevalence of lacunar infarcts and intracranial atherosclerotic thrombotic strokes, which are primarily linked to arterial sclerosis [7]. The BAD subtype of ischemic stroke is associated with a rapid progression of motor paralysis and a high likelihood of residual neurological deficits [3,8]. Additionally, as BAD-type infarction affects the territory of penetrating arteries, it is not amenable to endovascular therapy [9], necessitating a primarily medical approach using intravenous and oral pharmacological treatments. Intravenous thrombolysis (IVT) with alteplase, a recombinant tissue plasminogen activator, is an established treatment for acute ischemic stroke and is indicated within 4.5 hours of symptom onset, regardless of stroke subtype [9]. However, the efficacy of IVT in BAD-type infarction remains inconclusive. Furthermore, factors influencing the response to IVT in BAD-type stroke have yet to be fully elucidated.

Currently, the most effective treatment strategy for BAD remains unclear. To address this issue, we conducted a retrospective observational study to evaluate the efficacy of IVT for BAD. Furthermore, to gain deeper insights into the effects of IVT on BAD, we analyzed factors that may influence its effectiveness. Through these investigations, we aimed to determine whether IVT is a viable treatment strategy for BAD.

## Materials and methods

We conducted a single-center retrospective observational study of patients with BAD. The study included 98 patients who presented to the National Hospital Organization Disaster Medical Center, Tokyo, Japan, within 4.5 hours of symptom onset and were subsequently hospitalized between April 2010 and October 2024. Patients were included based on the following criteria: (1) those diagnosed with BAD in the lateral striate artery territory, characterized by a cerebral infarction larger than 15 mm, identified by high signal intensity on axial diffusion-weighted imaging (DWI) (slice thickness: 5-7 mm) on magnetic resonance imaging (MRI) or low signal density on axial computed tomography (CT) (slice thickness: 4-8 mm) and (2) those diagnosed with BAD in the paramedian pontine artery territory, defined by a vertically extending signal change along the sagittal plane from the ventral pons on axial MRI-DWI. Patients were excluded if they had ≥50% stenosis in the responsible major artery or developed atrial fibrillation or other cardiac embolic sources during hospitalization. All patients underwent at least one brain MRI and magnetic resonance angiography during their hospitalization for evaluation.

The 98 included patients were categorized into two groups: 11 patients who received IVT with alteplase (IVT group) and 87 patients who did not receive IVT (non-IVT group). Clinical outcomes were compared between the IVT and non-IVT groups. In the IVT group, the total dose of alteplase administered was 0.6 mg/kg, with 10% given as a rapid intravenous bolus and the remaining dose infused over 60 minutes [10]. Blood pressure was controlled below 180/105 mmHg for 24 hours following the administration of alteplase. No additional antithrombotic agents were administered within the first 24 hours following alteplase administration. Furthermore, within each group, patients were classified based on their functional outcomes at discharge using the modified Rankin Scale (mRS): those with an mRS of 0-2 were defined as the favorable outcome group, whereas those with an mRS of 3-6 were classified as the poor outcome group [11].

The clinical background of the patients included was analyzed, including age, sex, handedness, and family history of stroke. Stroke risk factors such as smoking, history of stroke, hypertension, dyslipidemia, diabetes, and cardiovascular disease were also assessed. Stroke severity at initial presentation was evaluated using the National Institutes of Health Stroke Scale (NIHSS) scores. Treatment modalities were recorded, including aspirin, clopidogrel, cilostazol, dual antiplatelet therapy, argatroban, and edaravone. Clinical outcomes, including admission period, NIHSS score at discharge, and mRS at discharge, were analyzed. In the IVT group, intracranial hemorrhage within 24 hours after administration of IVT was evaluated. Intergroup comparisons of these clinical factors were conducted.

Pairwise deletion was performed for missing values, and an available-case analysis was conducted. Fisher’s exact test was used to detect differences in the frequency of clinical background characteristics between the two groups. The Mann-Whitney U test was used to compare age, NIHSS scores, mRS scores, and length of hospital stay between the two groups, as well as the time to IVT administration within the IVT group. A p-value of <0.05 was considered statistically significant. All statistical analyses in this study were conducted using GraphPad Prism 9 software (GraphPad Software, San Diego, CA, USA).

## Results

**Figure 1:**
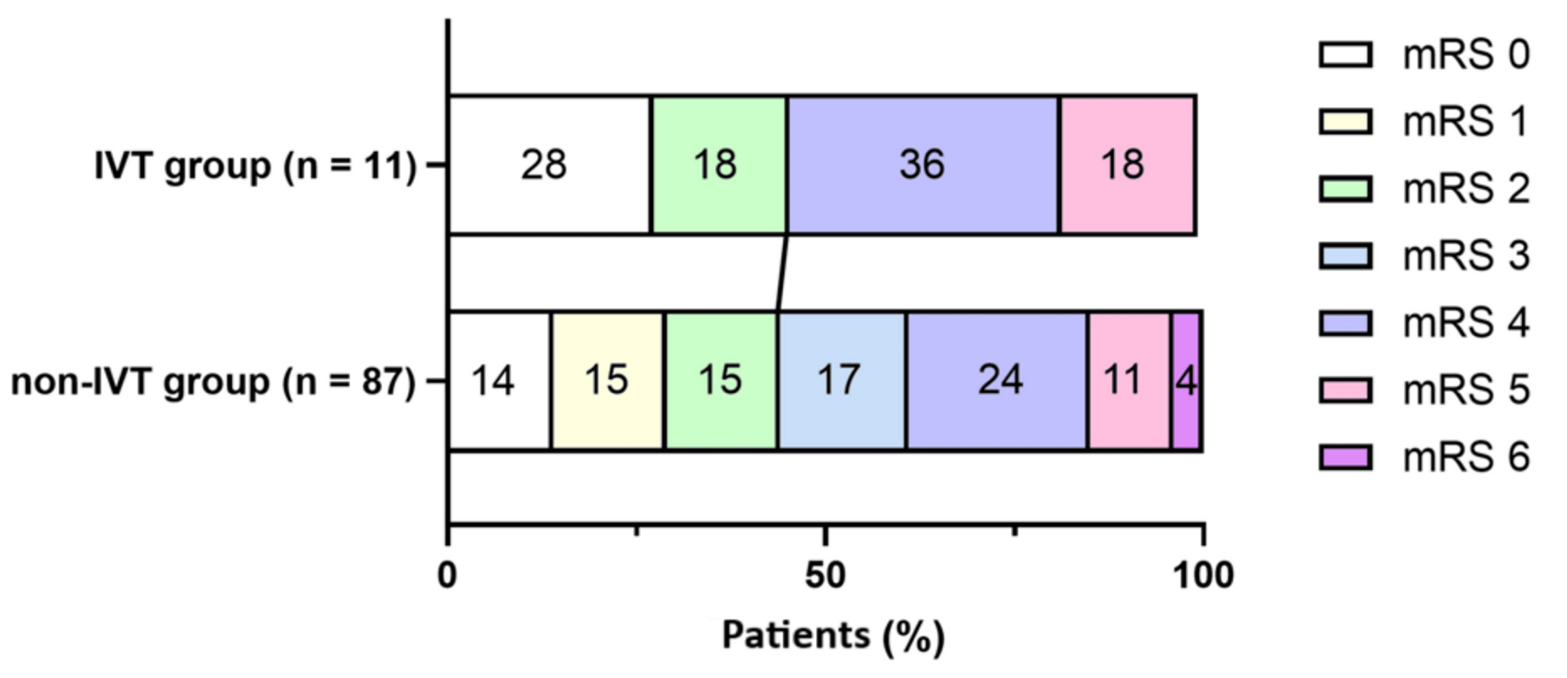
mRS at discharge in the IVT and non-IVT groups IVT, intravenous thrombolysis; mRS, modified Rankin Scale

First, the comparison between the IVT group and the non-IVT group was conducted. As for clinical background, patients in the IVT group were significantly younger than those in the non-IVT group (62.4 years vs. 75.4 years; p = 0.0003). However, aside from age, there were no significant differences in other clinical background factors, including sex, dominant hand, family history of stroke, or ischemic stroke risk factors. Regarding initial stroke evaluation, there were no significant differences in NIHSS scores at presentation between the IVT and non-IVT groups, suggesting that the initial stroke severity was generally comparable between the two groups. Lenticulostriate artery infarction accounted for 100% of cases in the IVT group and 73.6% in the non-IVT group, with no significant difference between the groups (p = 0.062). Regarding treatment, dual antiplatelet therapy was more frequently used in the IVT group than in the non-IVT group (p = 0.030), whereas the use of argatroban was significantly more frequent in the non-IVT group compared to the IVT group (p = 0.0002). As for clinical outcomes, there were no significant differences between the IVT and non-IVT groups in the admission period, NIHSS scores or mRS scores at discharge, nor in the proportion of patients classified as having a favorable prognosis. These results showed that there was no difference in initial stroke severity or discharge severity between the IVT and non-IVT groups. The clinical characteristics of the IVT and non-IVT groups are summarized in Table 1, and the mRS evaluation at discharge for each group is presented in Figure 1.

**Table 1:** Clinical characteristics of the IVT and non-IVT groups IVT, intravenous thrombolysis; mRS, modified Rankin Scale; NIHSS, National Institutes of Health Stroke Scale

Variables	IVT group (n = 11)	Non-IVT group (n = 87)	p-value	Test statistics
Clinical background				
Age (years; mean, SD)	62.4 (11.6)	75.4 (10.9)	0.0003	Mann-Whitney U test
Male sex	8/11 (72.7%)	57/87 (65.5%)	0.75	Fisher’s exact probability test
Dominant right hand	10/10 (100%)	56/59 (94.9%)	>0.99	
Family history of stroke	2/10 (20.0%)	5/68 (7.35%)	0.22	
Risk factor				
Smoking	7/11 (63.6%)	46/86 (53.5%)	0.54	Fisher’s exact probability test
Medical history of stroke	0/11 (0.0%)	21/78 (26.9%)	0.06	
Hypertension	7/11 (63.6%)	67/86 (77.9%)	0.28	
Hyperlipidemia	6/11 (54.5%)	49/85 (57.6%)	>0.99	
Diabetes	3/10 (30.0%)	32/83 (38.6%)	0.74	
Cardiovascular disease	0/11 (0.0%)	15/83 (18.1%)	0.20	
Initial stroke evaluation				
NIHSS at admission (mean, SD)	5.73 (3.55)	6.05 (5.51)	0.85	Mann-Whitney U test
The lenticulostriate artery infarction	11/11 (100%)	64/87 (73.6%)	0.062	Fisher’s exact probability test
The paramedian pontine artery infarction	0/11 (0%)	23/87 (26.4%)	0.062	
Treatment				
Aspirin	10/11 (90.91%)	62/87 (71.3%)	0.28	Fisher’s exact probability test
Clopidogrel	7/11 (63.6%)	41/87 (47.1%)	0.35	
Cilostazol	4/11 (36.4%)	15/87 (17.2%)	0.22	
Dual antiplatelet therapy	9/11 (81.8%)	41/87 (47.1%)	0.030	
Argatroban	2/11 (18.2%)	68/87 (78.2%)	0.0002	
Edaravone	8/11 (72.7%)	45/87 (51.7%)	0.21	
Outcome				
Favorable prognosis	5/11 (45.5%)	38/87 (43.7%)	>0.99	Fisher’s exact probability test
Admission period (days; mean, SD)	20.6 (10.8)	27.9 (40.6)	0.55	Mann-Whitney U test
NIHSS at discharge (mean, SD)	4.36 (4.15)	4.64 (5.26)	0.87	
mRS at discharge (mean, SD)	2.73 (2.00)	2.71 (1.72)	0.98	

Next, a subgroup analysis was conducted within the IVT group to compare patients with a favorable prognosis and those with a poor prognosis (Table 2). The favorable prognosis group with IVT included five patients, while the poor prognosis group comprised six patients. As for clinical background, patients in the favorable prognosis group were significantly younger than those in the poor prognosis group (53.4 years vs. 69.8 years; p = 0.0088). However, no significant differences were found between the two groups regarding other clinical background factors or ischemic stroke risk factors. Additionally, NIHSS and mRS scores at the initial visit did not significantly differ between the groups. The use of specific treatments other than IVT was comparable between the two groups. Furthermore, no patients exhibited intracranial hemorrhage on CT performed 24 hours after IVT administration. The analysis within the IVT group revealed that initial stroke severity did not differ between the favorable and unfavorable outcome groups, whereas younger age was associated with a favorable prognosis.

**Table 2:** Clinical characteristics of the favorable and poor prognosis groups with IVT *Intracranial hemorrhage was assessed by computed tomography 24 hours after IVT administration. IVT, intravenous thrombolysis; mRS, modified Rankin Scale; NIHSS, National Institutes of Health Stroke Scale

Variables	Favorable prognosis group with IVT (n = 5)	Poor prognosis group with IVT (n = 6)	p-value	Test statistics
Clinical background				
Age (years; mean, SD)	53.4 (6.88)	69.8 (9.04)	0.0088	Mann-Whitney U test
Male sex	4/5 (80.0%)	4/6 (66.7%)	>0.99	Fisher’s exact probability test
Dominant right hand	5/5 (100%)	6/6 (100%)	>0.99	
Family history of stroke	1/5 (20.0%)	1/3 (33.3%)	>0.99	
Risk factor				
Smoking	4/5 (80.0%)	3/6 (50.0%)	0.55	Fisher’s exact probability test
Medical history of stroke	0/5 (0.0%)	0/6 (0%)	>0.99	
Hypertension	3/5 (60.0%)	4/6 (66.7%)	>0.99	
Hyperlipidemia	4/5 (80.0%)	2/6 (33.3%)	0.24	
Diabetes	2/5 (40.0%)	1/5 (20.0%)	>0.99	
Cardiovascular disease	0/5 (0.0%)	0/6 (0%)	>0.99	
Initial stroke evaluation				
NIHSS score at admission (mean, SD)	5.80 (3.49)	5.67 (3.93)	0.95	Mann-Whitney U test
Time to IVT administration (minutes; mean, SD)	145.8 (34.1)	165.5 (43.5)	0.43	
The lenticulostriate artery infarction	5/5 (100%)	6/6 (100%)	>0.99	Fisher’s exact probability test
The paramedian pontine artery infarction	0/6 (0%)	0/6 (0%)	>0.99	
Treatment				
Aspirin	5/5 (100%)	5/6 (83.3%)	>0.99	Fisher’s exact probability test
Clopidogrel	3/5 (60.0%)	4/6 (66.7%)	>0.99	
Cilostazol	3/5 (60.0%)	1/6 (16.7%)	0.24	
Dual antiplatelet therapy	6/6 (100%)	4/6 (66.7%)	0.45	
Argatroban	1/5 (20.0%)	1/6 (16.7%)	>0.99	
Edaravone	5/5 (100%)	3/6 (50.0%)	0.18	
Outcome				
Admission period (days; mean, SD)	18.6 (15.4)	22.17 (6.01)	0.61	Mann-Whitney U test
NIHSS score at discharge (mean, SD)	0.4 (0.89)	7.67 (2.25)	<0.0001	
mRS score at discharge (mean, SD)	0.8 (1.10)	4.33 (0.52)	<0.0001	
Intracranial hemorrhage*	0/5 (0%)	0/5 (0%)	>0.99	Fisher’s exact probability t

Finally, to determine whether the comparative analysis results in the IVT group were unique to IVT-treated patients, a similar subgroup analysis was conducted within the non-IVT group (Table 3). The favorable prognosis group with non-IVT consisted of 38 patients, while the poor prognosis group with non-IVT included 49 patients. In the favorable prognosis group with non-IVT, patients were significantly younger than those in the poor prognosis group with non-IVT (70.8 years vs. 78.9 years; p = 0.0004). Moreover, patients in the favorable prognosis group with non-IVT had significantly lower NIHSS and scores at the initial visit, indicating a milder severity of stroke.

**Table 3:** Clinical characteristics of the favorable and poor prognosis groups with non-IVT IVT, intravenous thrombolysis; mRS, modified Rankin Scale; NIHSS, National Institutes of Health Stroke Scale

Variables	Favorable prognosis group with IVT (n = 38)	Poor prognosis group with IVT (n = 49)	p-value	Test statistics
Clinical background				
Age (years; mean, SD)	70.8 (8.68)	78.9 (11.2)	0.0004	Mann-Whitney U test
Male sex	32/38 (84.2%)	25/49 (51.0%)	0.0014	Fisher’s exact probability test
Dominant right hand	36/38 (94.7%)	31/33 (93.9%)	>0.99	
Family history of stroke	2/29 (6.90%)	3/23 (13.0%)	0.64	
Risk factor				
Smoking	27/41 (65.9%)	20/48 (41.7%)	0.033	Fisher’s exact probability test
Medical history of stroke	11/39 (28.2%)	12/43 (27.9%)	>0.99	
Hypertension	28/38 (73.7%)	39/48 (81.3%)	0.44	
Hyperlipidemia	27/37 (73.0%)	22/48 (45.8%)	0.015	
Diabetes	17/37 (45.9%)	15/46 (32.6)	0.26	
Cardiovascular disease	5/37 (13.5%)	10/46 (21.7%)	0.40	
Initial stroke evaluation				
NIHSS score at admission (mean, SD)	3.64 (2.55)	7.98 (6.42)	0.0002	Mann-Whitney U test
The lenticulostriate artery infarction	31/42 (73.8%)	35/49 (71.4%)	0.82	Fisher’s exact probability test
The paramedian pontine artery infarction	11/42 (26.1%)	14/49 (28.6%)	0.82	
Treatment				
Aspirin	27/37 (73.0%)	35/49 (71.4%)	>0.99	Fisher’s exact probability test
Clopidogrel	21/38 (55.3%)	20/49 (40.8%)	0.20	
Cilostazol	3/38 (7.89%)	12/49 (24.5%)	0.050	
Dual antiplatelet therapy	18/38 (47.4%)	22/49 (44.9%)	0.68	
Argatroban	32/38 (84.2%)	36/49 (73.5%)	0.30	
Edaravone	23/38 (60.5%)	22/49 (44.9%)	0.20	
Outcome				
Admission period (days; mean, SD)	23.9 (58.7)	31.1 (16.6)	0.41	Mann-Whitney U test
NIHSS score at discharge (mean, SD)	1.42 (1.77)	7.23 (5.72)	<0.0001	
mRS score at discharge (mean, SD)	1.03 (0.82)	4.02 (0.88)	<0.0001	

## Discussion

This study identified several important findings regarding the relationship between BAD and IVT. First, no significant improvement in prognosis was observed in the IVT group compared to the non-IVT group. Additionally, patients in the favorable prognosis group with IVT were significantly younger than those in the poor prognosis group with IVT. However, a similar trend was observed in the non-IVT group, where patients in the favorable prognosis group were also significantly younger than those in the poor prognosis group. These findings suggest that younger age may be a key factor influencing prognosis, regardless of IVT administration.

The effectiveness of IVT for BAD has been investigated in only three retrospective observational studies to date [5,12,13]. Deguchi et al. analyzed eight cases of BAD treated with alteplase (0.6 mg/kg) and reported that neurological symptoms worsened again in four of these patients [5]. Similarly, Park et al. examined nine BAD patients treated with alteplase (0.9 mg/kg) and found no improvement in prognosis compared to 26 BAD patients treated with antiplatelet therapy alone [12]. These two studies, consistent with our findings, suggest the limited efficacy of IVT for BAD. In contrast, Wu et al. compared 42 BAD patients treated with alteplase (0.9 mg/kg) to 42 patients receiving only antiplatelet therapy, with patient backgrounds standardized, and reported that the alteplase group had a better prognosis [13]. Wu et al. also suggested that the improved outcomes might be influenced by the higher alteplase dose (0.9 mg/kg) and the use of dual antiplatelet therapy [13]. However, in Japan, the approved alteplase dose is limited to 0.6 mg/kg, making its efficacy for BAD potentially less promising. Furthermore, a key limitation of alteplase-based treatment strategies is the restriction on using additional antithrombotic agents within the first 24 hours post-administration [9]. Since platelet activation has been implicated in the progression of neurological symptoms in BAD [14], antiplatelet therapy is considered a crucial component of treatment [3]. The 24-hour restriction on antiplatelet therapy may contribute to the limited prognostic benefits of alteplase in BAD. Similarly, due to concerns about bleeding risk, blood pressure must be controlled below 180/105 mmHg for 24 hours following the administration of alteplase [9]. Previous studies have emphasized the importance of maintaining collateral blood flow via microcirculation from neighboring lenticulostriate arteries to prevent the expansion of lenticulostriate artery territory infarcts [15]. This suggests that blood pressure management after alteplase administration may contribute to the worsening of neurological symptoms in BAD.

These findings highlight the need for more effective treatment strategies for BAD. Currently, tenecteplase, an alternative IVT agent to alteplase, is gaining attention due to its longer half-life and higher fibrin specificity compared to alteplase [3]. Clinical trials have demonstrated its favorable efficacy on acute ischemic stroke [16,17], and its effectiveness in BAD should be further investigated. Additionally, in addition to platelet function, the role of fibrin thrombi resulting from significant increases in coagulation and fibrinolysis activation is believed to contribute to the progression of BAD [3]. Therefore, anticoagulants such as argatroban are expected to play a role in the treatment of BAD. A treatment strategy involving the early initiation of argatroban following alteplase administration has been explored in a previous clinical trial, but its efficacy has not been established [18]. However, its potential benefits in BAD should be specifically evaluated. Notably, no cases of intracranial hemorrhage were observed in our study, and previous reports also suggest a low risk of bleeding associated with alteplase use in BAD [5,12,13]. Therefore, the optimal timing for initiating antiplatelet therapy after alteplase administration in BAD warrants further discussion, including the possibility of earlier initiation.

To the best of our knowledge, this study is the first to investigate factors influencing the efficacy of IVT in BAD and to identify age as a significant factor affecting its effectiveness. A previous study reported that younger patients receiving alteplase tend to have better outcomes [19]. However, younger age is generally associated with a favorable prognosis in ischemic stroke, regardless of alteplase administration [20], as observed in both the IVT and non-IVT groups in this study. Additionally, a recent study has reported that the initial NIHSS score is not a reliable prognostic factor for BAD [21]. Since motor paralysis in BAD tends to fluctuate, the initial severity may not accurately reflect disease progression. In our study, there was no significant difference in initial NIHSS scores between the favorable and poor prognosis IVT groups, which is consistent with previous findings. Consequently, a more detailed analysis with a larger patient cohort is necessary to clarify the prognostic factors of IVT for BAD.

This study has several limitations. First, as a retrospective observational study, patient backgrounds were not uniformly distributed across groups. Notably, argatroban was used more frequently in the non-IVT group. However, its use in BAD patients who did not receive IVT was clinically reasonable, making it challenging to standardize treatment protocols aside from IVT. Therefore, the study effectively compares clinical outcomes between IVT recipients and those receiving the best available treatment without IVT. Nevertheless, since no significant differences were observed between the IVT and non-IVT groups in terms of initial stroke severity or clinical background factors other than age, IVT did not demonstrate a prognostic benefit. This finding reinforces the notion that IVT may have limited effectiveness in improving outcomes for BAD. Second, the IVT group included only 11 cases, highlighting the limitation of a small sample size. However, subgroup analysis revealed that patients with a favorable prognosis in the IVT group were significantly younger than those with a poor prognosis, underscoring the potential impact of age on IVT outcomes. Third, this study did not assess mRS at a standardized time point (either three or six months after onset) as a long-term outcome measure or evaluate early neurological deterioration, which has recently been recognized as a key factor in BAD management [3]. Fourth, in this study, the decision to administer IVT was influenced by the attending physician’s judgment, which may have introduced selection bias between patient groups. Consequently, this could have resulted in a smaller number of patients in the IVT group, potentially limiting the statistical power of the analysis. Future prospective randomized controlled trials with larger sample sizes are needed to evaluate the efficacy of IVT in BAD thoroughly. Additionally, potential biomarkers and imaging modalities that may aid in stratifying BAD patients who could benefit from IVT should also be explored.

## Conclusions

Our study findings provide two important insights into the management of BAD. First, our comparison of the IVT and non-IVT groups revealed no significant improvement in prognosis with IVT treatments. Second, in both groups, patients with a favorable prognosis were significantly younger than those with a poor prognosis, highlighting age as a crucial prognostic factor in ischemic stroke. Although the efficacy of IVT remains uncertain, no cases of intracranial hemorrhage were observed after its administration, suggesting that IVT is a safe treatment option for BAD. A prospective randomized controlled trial to evaluate the efficacy of IVT for BAD, along with investigations into alternative treatment approaches, is needed. Additionally, a more detailed analysis with a larger patient cohort is necessary to clarify the prognostic factors of IVT for BAD.
